# Leucosceptosides A and B: Two Phenyl-Ethanoid Glycosides with Important Occurrence and Biological Activities

**DOI:** 10.3390/biom12121807

**Published:** 2022-12-02

**Authors:** Claudio Frezza, Daniela De Vita, Chiara Toniolo, Fabio Sciubba, Lamberto Tomassini, Alessandro Venditti, Armandodoriano Bianco, Mauro Serafini, Sebastiano Foddai

**Affiliations:** 1 Dipartimento di Biologia Ambientale, Università di Roma “La Sapienza”, Piazzale Aldo Moro 5, 00185 Rome, Italy; 2 NMR Lab, Università di Roma “La Sapienza”, Piazzale Aldo Moro 5, 00185 Rome, Italy; 3 Dipartimento di Chimica, Università di Roma “La Sapienza”, Piazzale Aldo Moro 5, 00185 Rome, Italy

**Keywords:** leucosceptoside A, leucosceptoside B, occurrence in plants, chemophenetics, biological activities

## Abstract

In this review paper, the occurrence in the plant kingdom, the chemophenetic value and the biological activities associated with two specific phenyl-ethanoid glycosides, *i.e.*, leucosceptoside A and leucosceptoside B, were reported. This is the first work ever conducted on such a subject. Analysis of the literature data clearly led to three important conclusions: leucosceptoside A is much more common in plants than leucosceptoside B; leucosceptoside A exerts more biological activities than leucosceptoside B even if nothing can be generally concluded about which one is actually the most potent; neither of these compounds can be used as a chemophenetic marker. These three aspects and more are discussed in more depth in this work.

## 1. Introduction

Leucosceptoside A and leucosceptoside B are two natural compounds belonging to the class of phenyl-ethanoid glycosides which have gained increasing attention in recent years due to their important and effective biological activities [1]. 

Phenyl-ethanoid glycosides are biosynthetically generated merging two different processes, the shikimic acid pathway, which produces the β-hydroxy-tyrosol part of phenyl-ethanoid glycosides, and the cinnamate pathway, which produces its caffeic acid part after a series of intermediate steps [2].

**Figure 1:**
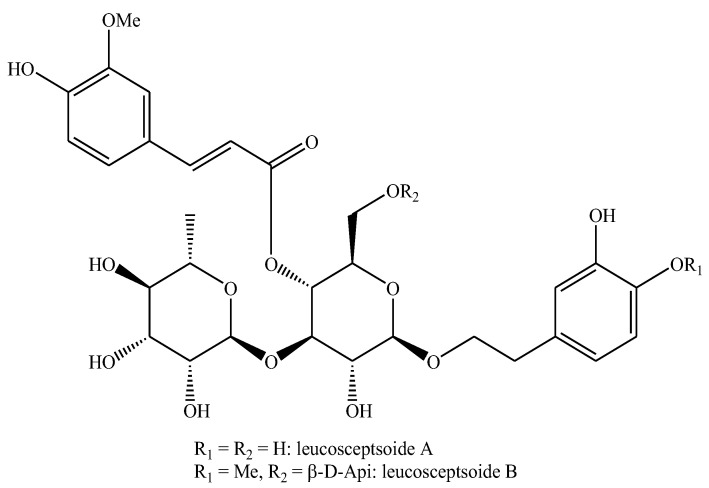
Structures of leucosceptoside A and leucosceptoside B.

The base compound of phenyl-ethanoid glycosides is called verbascoside, acetoside or acteoside, which is structurally characterized by a central glucose residue linked to one β-hydroxy-tyrosol and one rhamnose residue through ether bonds, and to one caffeic acid through an ester bond. Leucosceptoside A differs from verbascoside for the substitution with a methyl group in the caffeic acid moiety. Indeed, leucosceptoside B differs from verbascoside for the substitution with one methyl group each in the caffeic acid and β-hydroxy-tyrosol moieties as well as for the substitution with one β-D-apiose residue in the position 6 of the glucose moiety [3] (Figure 1). 

In the literature, there are some review papers on phenyl-ethanoid glycosides in general, exploring mainly their occurrence, structure and biological activities [1,2,4]; however, no review paper has ever focused its complete attention on leucosceptoside A and leucosceptoside B, which have often been actually neglected regarding several of their aspects in the past reviews. In addition, some of these reviews are now outdated and do not cover the chemophenetics of these compounds or even of the phenyl-ethanoid glycosides in general. These represent the main reasons why this review paper was written. In this, the specific occurrence of leucosceptoside A and leucosceptoside B in the plant kingdom as well as their relative biological activities were reported with major details. In addition, chemophenetic conclusions on these compounds were drawn and a strict comparison on the biological activities of these two compounds in more senses was also performed, both for the first time. This review paper is based on all the published papers until now, as recovered from Scopus, Reaxys, Google Scholar and PubMed. Only works regarding plants were considered, thus, excluding cell cultures. In addition, only works without any kind of procedure possibly producing alterations and artifacts in the phytochemical pattern were cited. Lastly, papers not written in English were not considered. 

## 2. Occurrence in the Plant Kingdom 

In the following Tables, the occurrences of leucosceptoside A (Table 1) and leucosceptoside B (Table 2) in the plant kingdom are reported and divided according to the family of the plant they have been isolated from. These tables are sub-divided according to the plant species and report data on the collection area of the plant cited, the studied organs and the methodologies of isolation and identification for these compounds. In the collection area columns, if not differently specified, the studied population or populations are intended to be collected from the wild. 

Leucosceptoside A proved to be quite common in the plant kingdom. In fact, it has been evidenced in eleven families. In particular, its lowest occurrence was observed in Asteraceae, Malvaceae and Oleaceae with one report each. In contrast, the highest occurrence has been noticed in the Lamiaceae family with 69 reports even though these were singular reports in most cases. Nevertheless, it is important to underline that not all the genera in the Lamiaceae family have shown the presence of this compound. Within the Lamiaceae family, this compound has been found particularly in the *Phlomis* L. genus with 15 reports whereas it is quite rare in *Betonica* L., *Schnabelia* Hand.-Mazz. and *Volkameria* L. genera with only one report of its presence each. Henceforth, further phytochemical studies on species belonging to these genera are necessary in order to verify if this compound is really part of their phytochemical components or if its presence was only accidental. All the other seven families see a moderate occurrence of leucosceptoside A. For what concerns the collection areas of the studied species, no significatively restricted zone for the occurrence of this compound has been observed since its presence has been found in species collected from four continents, *i.e.*, America, Europe, Africa and Asia. Nothing can be stated at the moment about the Oceanian situation since no work on it has been found. As for the studied organs, leucosceptoside A has shown no particular preference for a specific accumulation site having been individuated in leaves, aerial parts, tubers, roots and the whole plant even if it seems this compound prefers aerial organs. A few procedures have been used to extract this compound mainly associated with maceration processes in their different forms. Indeed, several techniques have been used for its isolation and identification, including high performing liquid chromatography relative techniques, liquid chromatography relative techniques, thin-layer chromatography relative techniques, infrared, ultraviolet and nuclear magnetic resonance spectroscopies and mass spectrometry. 

In the end, it is extremely relevant to highlight that Table 1 clearly indicates how leucosceptoside A cannot be used as a chemophenetic marker at any level since it has shown no specificity and also because the families where it was found are taxonomically very distant from each other. 

With respect to leucosceptoside A, leucosceptoside B proved to be less common in the plant kingdom. In fact, it has been evidenced only in six families. Its lowest occurrence was observed in the Bignoniaceae, Orobanchaceae, Plantaginaceae and Verbenaceae families with one report each, whereas the highest occurrence has been noticed in the Lamiaceae family again with seventeen reports. There is a certain parallelism between the results found for leucosceptoside A and those found for leucosceptoside B for what concerns the collection areas of the studied species, the studied organs and the methodologies adopted for their isolation and identification. Indeed, there are some important differences for what concerns the families, genera and species where their presence has been reported. In particular, leucosceptoside B has not been reported in the Acanthaceae, Compositae, Linderniaceae, Malvaceae and Oleaceae families. In addition, for what concerns the Bignoniaceae, Scrophulariaceae and Verbenaceae families, leucosceptoside B has been found in completely different species, if not genera themselves, than leucosceptoside A. For what concerns the Lamiaceae family, the reported genera and species are not generally the same as for leucosceptoside A. In particular, leucosceptoside B has not been found in the *Betonica* L., *Caryopteris* Bunge, *Clerodendrum* L., *Galeopsis* L., *Lagopsis* Bunge, *Leonurus* L., *Salvia* L., *Scutellaria* L., *Sideritis* L. and *Volkameria* L. genera. This situation prompts the need to perform more phytochemical analyses on the species for its further research, also because several singular reports have been found in the literature with the same conclusions as drawn before. 

Leucosceptoside B cannot also be used as a chemophenetic marker at any level for the same reasons as presented before, even if it presents a lower occurrence than leucosceptoside A. 

## 3. Biological Activities

### 3.1. Leucosceptoside A 

Leucosceptoside A has shown several interesting pharmacological activities. In the following lines, these activities are explored and detailed one-by-one. 

#### 3.1.1. Antioxidant and Radical Scavenging Activity

Leucosceptoside A proved to be a good antioxidant compound. Several studies against DPPH.^+^ confirm this with quite similar efficacy values. In particular, Ersöz et al. [58] obtained an IC_50_ value of 76.0 µM, which is lower than ascorbic acid used as a positive control (IC_50_ = 112 µM). Harput et al. [7] observed an IC_50_ value of 18.43 μg/mL, which is higher than quercetin but comparable (IC_50_ = 4.3 µg/mL), whereas Ono et al. [154] obtained an EC_50_ value of 25.7 µM, which is very similar to that for α-tocopherol (EC_50_ = 25.9 µM). Huang et al. [150] obtained an IC_50_ value of 72.14 μM, which is lower than vitamin C (IC_50_ = 81.83 µM). Lan et al. [117] obtained an EC_50_ value of 11.26 μM, which is slightly better than the positive control, ascorbic acid (EC_50_ value of 12.29 μM). Indeed, Calis et al. [60] obtained an IC_50_ value of 125.4 µM, which is quite higher than that of *dl*-α-tocopherol, used as a positive control (IC_50_ = 75.5 µM). Delazar et al. [61] obtained an RC_50_ of 0.0148 mg/mL, which is higher than trolox and quercetin, used as positive controls (RC_50_ values of 0.00307 and 0.0000278 mg/mL), instead. Lastly, Shen et al. [13] obtained an IC_50_ value of 53.32 µM, which was found to be higher than that of BHT, but no value of this latter was given. 

On the other hand, Charami et al. [86] expressed the results as percentages of inhibition, which were 88.3% and 89.0% after 20 and 60 min, respectively, at the concentration of 0.1 mM, whereas the positive control acetyl salicylic, at the same concentration and times, showed a percentage of inhibition of 80.6%. Additionally, Harput et al. [54] expressed their results as percentages of inhibition and compared their results with three positive controls. In particular, the value for leucosceptoside A was 41.8% at the concentration of 200 µM, which is near to ascorbic acid and BHA (percentages of inhibition equal to 39.9 and 35.6%, respectively) but worse than chlorogenic acid (percentage of inhibition equal to 68.6%). 

#### 3.1.2. Radical Scavenging

Leucosceptoside A was observed to be a modest radical scavenging compound. In particular, Wang et al. [112] studied the scavenging properties of this compound with respect to superoxide anion and obtained a SC_50_ of 0.294 mM, which is higher than all the other phenyl-ethanoid glycosides studied in this work. The best one was verbascoside, which presents an SC_50_ value of 0.063 mM. In addition, Wang et al. [112] also studied the antioxidant potential of this compound through a luminol-enhanced chemiluminescence assay against *N*-formyl-methionyl-leucyl-phenylalanine in stimulated human polymorphonuclear neutrophils. The results showed that this compound is a weak inhibitor of hydroxyl radicals with a percentage of inhibition of 27.8% at the concentration of 0.55 mM and a modest iron reductor with a percentage of 17.86% at the concentration of 1.57 mM. The former value represents the lowest percentage of inhibition among all the phenyl-ethanoid glycosides considered in this study, whereas the latter value is the second worst. Verbascoside was the most active as an inhibitor of the hydroxyl radical with a percentage of 55.7% at the concentration of 0.55 mM, whereas pedicularioside M was the most active as an iron reductor with a percentage of 23.57% at the concentration of 1.73 mM. Heilmann et al. [185] also studied the radical scavenging effects of this compound in *N*-formyl-methionyl-leucyl-phenylalanine-stimulated human polymorphonuclear neutrophils through a luminol-enhanced chemiluminescence assay. The obtained IC_50_ value was 0.18 µM, which is higher but not too far from that of quercetin used as a positive control (IC_50_ value = 0.5 µM). Nevertheless, most of the other phenyl-ethanoid glycosides studied in this work showed better results than leucosceptoside A and, in particular, echinacoside was the best one with an IC_50_ value of 0.03 µM. 

#### 3.1.3. Anti-Inflammatory Activity

Leucosceptoside A shows moderate anti-inflammatory activities with respect to NO production. In particular, Han et al. [151] obtained an IC_50_ value of 9.0 µM in the Raw 264.7 cell line, whereas the positive control aminoguanidine had an IC_50_ value of 10.7 µM. Vien et al. [14] obtained a different result in LPS-stimulated BV2 microglial cells using the Griess assay, with an IC_50_ value of 61.1 µM, which is much higher than the positive control butein (IC_50_ = 4.5 µM). In contrast, Ochi et al. [147] obtained a moderate percentage of inhibition of 40% in cell culture supernatants of LPS-stimulated RAW264.7 macrophages at the concentration of 100 µM, whereas the positive control L-NMMA presents an inhibition value of 55% at the same concentration. 

#### 3.1.4. Enzyme Inhibitory Activity 

Leucosceptoside A has been also studied for its potential inhibitory effects on some enzymes, *i.e.*, α-glucosidase, acetylcholinesterase, protein kinase C alpha and tyrosinase. 

Concerning α-glucosidase, this compound has shown contrasting results. In fact, Liu et al. [27] obtained an IC_50_ value of 0.7 mM, which is much lower than acarbose used as a positive control (IC_50_ = 14.4 mM). Indeed, Thu et al. [146] obtained an IC_50_ value of 273.0 µM, which is a little higher than that of acarbose (IC_50_ = 204.2 µM). 

Leucosceptoside A also possesses between moderate and modest acetylcholinesterase inhibitory effects. In fact, Kang et al. [33] obtained an IC_50_ value of 423.7 µg/mL, which is much higher than Captopril^®^ used as a positive control (IC_50_ value of 20 nM). In addition, Saidi et al. [186] obtained an IC_50_ value of 72.85 µM, which is also much higher than that of the positive control used in this study, *i.e.*, galantamine (IC_50_ value of 4.14 µM). Indeed, Li et al. [187], using the Scheffe’s test, obtained an IC_50_ value of 3.86 mM, which is comparable to that of Captopril^®^ used again as a positive control (IC_50_ value of 2.11 nM). 

This compound is also a modest protein kinase C alpha inhibitor with an IC_50_ value of 19.0 µM. This value is good in number but, in the same study, other phenyl-ethanoid glycosides were found to be more active, such as forsythoside and verbascoside, showing IC_50_ values of 4.6 and 9.3 µM, respectively [131]. 

In addition, this compound shows only modest tyrosinase inhibitory activities, with a percentage of inhibition of 21.65% at the concentration of 0.051 mM. Kojic acid, used as a positive control, has a percentage of inhibition of 53.87% at the concentration of 0.047 mM [188]. A similar result was obtained by Saidi et al. [186], who observed a percentage of inhibition of 39.5% at the concentration of 100 µM, whereas the positive control hydroquinone has a percentage of inhibition of 72.0% at the same concentration.

Lastly, this compound has shown no effect on the hyaluronidase enzyme inhibition test and collagenase enzyme inhibition test at the concentration of 100 µg/mL [138].

#### 3.1.5. Hepatoprotective Activity 

At 100 µM, leucosceptoside A exerts strong hepatoprotective properties in pretreatment as obtained by studying the viability of HepG2 cells after CCl_4_ intoxication, by means of an MTT assay and flow cytometry, with values of cell number and cell viability both above 80%, thus, implying a restoration of cell survival. These values are extremely good in numbers, but no standard compound was tested for comparison. The mechanism of action occurs through the inhibition of NF-kB activation since it was observed that pretreatment with this compound can inhibit CCl_4_-induced lipid peroxidation in HepG2 cells by 177.73% and attenuate the decrease in SOD by 76.58%; furthermore, pre-incubation of the same cells with this compound can prevent ROS production by 183.56% [13]. 

#### 3.1.6. Neuroprotective Activity 

Leucosceptoside A, in pretreatment, exerts good neuroprotective effects against the 1-methyl-4-phenylpyridinium ion (MPP^+^)-induced cell death in mesencephalic neurons of rats. MPP^+^ is the ion produced after the biotransformation of the neurotoxin 1-methyl-4-phenyl-1,2,3,6-tetrahydropyridine by the monoamine oxidase B, and it is well known to cause Parkinson’s disease in rats and non-human primates. By means of an MTT assay, it was observed that leucosceptoside A, at the concentration of 4 µM, reduces cell death by 7%, whereas at the concentration of 16 µM, it increases cell growth by 3.7%. These values are extraordinary, however, in the same study, a more efficient compound was found, *i.e.*, pedicularioside A. In fact, at the concentration of 4 µM, this latter compound shows a reduction value of cell death by 3.4% and, at the concentration of 16 µM, it increases cell growth by 21.4% [189].

#### 3.1.7. Cytoprotective Activity 

Leucosceptoside A exhibits promising cytoprotective effects against *t*-BHP-induced toxicity in HepG2 cells with an IC_50_ value of 21.1 µM. This value is lower than silymarin used as a positive control (IC_50_ = 37.1 µM) [23]. 

#### 3.1.8. Anticomplementary Activity 

Leucosceptoside A exerts good anticomplementary effects with a CH_50_ value of 0.23 mM, which is slightly higher than that of the standard compound heparin (CH_50_ = 0.06 mM) [119].

#### 3.1.9. Anti-HIV Activity 

Leucosceptoside A has shown strong HIV-1 integrase inhibitory effects with an IC_50_ value of 29.4 µM. This value is better than curcumin (IC_50_ = 54.3 µM) and comparable to L-chicoric acid (IC_50_ = 21.0 µM) used as positive controls [32]. 

#### 3.1.10. Cytotoxic Activity 

According to Argyropoulou et al. [46], leucosceptoside A exerts very modest cytotoxic effects on HeLa (human epithelial carcinoma), MCF7 (breast metastatic adenocarcinoma) and HC7-116 (colon cancer) cell lines with IC_50_ values of 200, 189.08 and 182.33 µg/mL, respectively. The standard compound doxorubicin presents IC_50_ values of 0.175, 0.235 and 0.096 µg/mL, respectively, whereas the other standard compound actinomycin D presents IC_50_ values of 0.013, 0.022 and 0.019µg/mL, respectively. On the contrary, it showed no effect against melanoma. Saidi et al. [186] also tested the cytotoxic effects of this compound on HeLa cell lines obtaining an IC_50_ = 80.87 µM as well as a positive result on the A549 (adenocarcinomic human alveolar basal epithelial cells) cancer cell line obtaining an IC_50_ = 99.00 µM. All these values are much higher than the standard compounds used, *i.e.*, doxorubicin for the former cancer cell line (IC_50_ = 0.36 µM) and ellipticine for the latter cancer cell line (IC_50_ = 0.31 µM). Abe et al. [159] obtained promising results on Hela (GI_50_ = 42 µM) and also against the B16F10 (murine melanoma) cell line with a GI_50_ value equal to 28 µM and against the MK-1 (gastric carcinoma with liver metastasis) cell line with a GI_50_ value equal to 33 µM. All these values are good in numbers, but some other phenyl-ethanoid glycosides studied in this work present better results in full or in part. In particular, *iso*-verbascoside was the most potent against B16F10 and MK-1 with GI_50_ values of 10 and 32 µM, respectively, whereas arenarioside was the most potent against HeLa with a GI_50_ value of 34 µM. Unlike the previous works, Saracoglu et al. [49] did not observe any effect on HeLa cells for this compound and this contrast all depends on the methodology adopted. Saracoglu et al. [49] did not observe any effect against dRLh-88 (rat hepatoma), P-388-d1 (mouse lymphoid neoplasma) and S-180 (sarcoma) cancer cell lines, too. 

#### 3.1.11. Inhibitory Activities 

Leucosceptoside A exhibits good inhibitory effects against ADP + NADPH-induced lipid peroxidation in rat liver microsomes with an IC_50_ value of 1.69 µM, which is very promising. Yet, other phenyl-ethanoid glycosides studied in this work showed better results. In particular, *iso*-verbascoside was the most potent with an IC_50_ value of 0.38 µM [132]. 

This compound also exhibits inhibitory effects on the protonation of thymine radical anion induced by pulse radiolysis, which causes severe damages in cells with a reaction rate constant of electronic transfer equal to 1.54 × 10^9^ dm^3^ mol^−1^ s^−1^. This value is very good in number, but without a direct comparison with any compound [190]. 

#### 3.1.12. Antimicrobial Activities 

Leucosceptoside A exerts poor antimicrobial effects against *Staphylococcus aureus* ATCC29213 and *Enterococcus faecalis* ATCC29212 with MIC values of 1000 µg/mL [60]. These values are extremely high and are generally considered to be inactive. 

Indeed, it has shown no activity against *Escherichia coli* ATCC25922 [60], *Mycobacterium tuberculosis* H37Rv [30], *Pseudomonas aeruginosa* ATCC27853 [60], *Bacillus subtilis* NBRC3134 and *Klebsiella pneumoniae* NBRC3512 [24].

#### 3.1.13. Antifungal Activities 

Leucosceptoside A has shown no activity against *Candida albicans* ATCC90028, *Candida krusei* ATCC6258 and *Candida parapsilosis* ATCC22019 [60].

### 3.2. Leucosceptoside B 

Leucosceptoside B has also shown some interesting pharmacological activities, even if less numerous than leucosceptoside A. In the following lines, these activities are explored and detailed one-by-one. 

#### 3.2.1. Antioxidant Activity

Leucosceptoside B proved to be an antioxidant compound. Several studies, according to different assays, confirm this with opposite efficacy values. 

According to the DPPH.^+^ assay, this compound exerts between moderate and modest effects. In fact, Niu et al. [166] obtained an IC_50_ value of 31.16 µM, which is higher than ascorbic acid used as a positive control (IC_50_ = 7.81 µM). In addition, Georgiev et al. [182] obtained an IC_50_ value of 96 µg/mL, which is high with respect to the other phenyl-ethanoid glycosides studied in this work. In particular, this compound was found to be the weakest, whereas the most potent was forsythoside B with an IC_50_ value of 21 µg/mL. Indeed, Lan et al. [117] obtained an EC_50_ value of 13.05 μM, which is comparable to that of ascorbic acid (EC_50_ value of 12.29 μM). Lastly, Calis et al. [60] obtained an IC_50_ value of 61.3 µM, which is lower than that of *dl*-α-tocopherol used as a positive control (IC_50_ = 75.5 µM); yet there was one phenyl-ethanoid glycosides (myricoside) that is even more potent than this (IC_50_ = 46.8 µM).

#### 3.2.2. Radical Scavenging Activity

Through a luminol-enhanced chemiluminescence assay with formyl-methionyl-leucyl-phenylalanine in stimulated human polymorphonuclear neutrophils, Heilmann et al. [185] reported the radical scavenging activities of this compound with an IC_50_ value of 0.17 µM, which is higher but not too far from that of quercetin used as a positive control (IC_50_ value = 0.5 µM). 

Indeed, Georgiev et al. [182] also studied the ORAC, HORAC, FRAP and superoxide anion activities of this compound obtaining different results. In particular, good effects were observed for the first one with a value of 16264.7 ORACFL/g, which is higher than chlorogenic acid used as a standard compound (9172.8 ORACFL/g), and for the second one with a value of 3885.1 HORACFL/g, which is higher than chlorogenic acid used as a standard compound (3584.5 ORACFL/g). Conversely, poor effects were observed for the third assay with a value of absorbance of 0.602 at 700 nm, which is much lower than chlorogenic acid used as a reference compound (3.547), whereas moderate effects were observed for the last assay with an IC_50_ value of approximately 45 µg/mL, which is much higher with respect to the other phenyl-ethanoid glycosides studied in this work, and in particular, of verbascoside, which was the best one (IC_50_ = 4.2 µg/mL). 

#### 3.2.3. Anti-Inflammatory Activity

Leucosceptoside B exerts good inhibitory effects on cobra venom factor-induced alternative pathway activation in mouse sera with a percentage of inhibition of 40% and on COX-2 production with a percentage of inhibition near 30%. The positive control, indomethacin, presents a percentage of inhibition near 70%. In addition, it shows moderate inhibitory effects on NO production and IL-10 production as well as good inhibitory effects on COX-1, even if in the study, the values were not clearly expressed. Furthermore, all these values are higher than indomethacin [183]. 

#### 3.2.4. Enzyme Inhibitory Activity

Georgiev et al. [182] studied the acetylcholinesterase and butyrylcholinesterase inhibitory activities of leucosceptoside B. In both cases, the activity is dose-dependent; however, in the former, the percentage of inhibition is almost 50% at the concentration of 100 µg/mL, whereas in the latter, the percentage of inhibition is a little above 10% at the concentration of 100 µg/mL. These values are much lower than the positive control galanthamine, which presents percentages of inhibition of 98.97 and 89.95%, respectively, at the same concentrations. 

Indeed, Cespedes et al. [191] obtained a different result for the acetylcholinesterase inhibitory effects of this compound with an IC_50_ value of 20.1 µg/mL, whereas galanthamine has an IC_50_ value of 13.2 µg/mL. 

Cespedes et al. [191] did not observe any butyrylcholinesterase inhibitory activity for leucosceptoside B.

#### 3.2.5. Neuroprotective Activity

Leucosceptoside B, in pretreatment, is also able to exert strong neuroprotective effects against the 1-methyl-4-phenylpyridinium ion (MPP^+^)-induced cell death in mesencephalic neurons at the concentration of 40 µg/mL. The potency was measured as optical density with a value of 1.06. However, buddleoside A, another phenyl-ethanoid glycoside, was found to be much more potent with a value of 1.62 [149].

#### 3.2.6. Inhibitory Activities

Leucosceptoside B has shown no human lactate dehydrogenase inhibitory activity [172]. 

#### 3.2.7. Antimicrobial Activity

Leucosceptoside B has shown very poor antimicrobial effects against *Staphylococcus aureus* ATCC29213 and *Enterococcus faecalis* ATCC29212 with MIC values of 1000 µg/mL [60]. Again, these values are extremely high and are generally considered to be inactive. 

In addition, it has proven to be inactive against *Escherichia coli* ATCC25922 and *Pseudomonas aeruginosa* ATCC27853 [60].

#### 3.2.8. Antimicrobial Activity

Leucosceptoside B has shown no antifungal activity against *Candida albicans* ATCC90028, *Candida krusei* ATCC6258 and *Candida parapsilosis* ATCC22019 [60].

### 3.3. Comparing the Biological Results of Leucosceptosides A and B

Table 3 and Table 4 below summarize the biological activities and efficacy values of leucosceptoside A and leucosceptoside B, respectively.

According to the data as reported in Section 3.1 and Section 3.2 and Table 3 and Table 4, leucosceptoside A proved to be a more important biological compound than leucosceptoside B based on the number of biological assays performed on them. In fact, leucosceptoside A has been tested for more numerous biological assays than leucosceptoside B (10 vs. 5). In particular, leucosceptoside A was also tested for its hepatoprotective, cytoprotective, cytotoxic, anticomplementary and anti-HIV activities with respect to leucosceptoside B. This minor number of assays performed on leucosceptoside B may be explained not only by its minor occurrence in the plant kingdom but also for the fact that this compound is much more difficult to isolate in pure form since its co-elution with more polar compounds than phenyl-ethanoid glycosides is extremely common. On the contrary, given the literature data results, it is not so easy to ascertain which compound is generally the most efficient. This is due to the fact that leucosceptoside A and leucosceptoside B were directly compared only in two cases. The first case regards the radical scavenging effects of these compounds in formyl-methionyl-leucyl-phenylalanine-stimulated human polymorphonuclear neutrophils through a luminol-enhanced chemiluminescence assay and showed that leucosceptoside A and leucosceptoside B basically have the same efficacy (IC_50_ values of 0.18 µM *vs.* 0.17 µM) [185]. The second case regards the DPPH.^+^ radical scavenging assay and showed that leucosceptoside A is slightly better than leucosceptoside B (EC_50_ values of 11.26 µM *vs*. 13.05 µM) [117]. One further indirect comparison of the efficacy values of these two compounds can be made also according to the DPPH.^+^ radical scavenging assay; however, the relative studies led to extremely contrasting results. In fact, the IC_50_ values found for leucosceptoside A were 76.0 µM [58], 18.43 µM [7], 72.14 µM [150], 125.4 µM [60] and 53.32 µM [13], whereas the IC_50_ values found for leucosceptoside B were 31.16 µM [166], 96.0 µM [182] and 61.3 µM [60]. These discrepancies are due to several intrinsic methodological factors and are also dependent on the fact that leucosceptosides A and B were not studied together in these works. In this context, these results do not really allow to make any general conclusion on which of these compounds is the most potent. Indeed, for what concerns the rest of the biological activities, no comparison can be made since the two compounds were not studied together, different protocols were used, and the values were expressed in different unities of efficacy. These last aspects are certainly something to keep in consideration for future research in order to improve this situation. 

## 4. Conclusions

This review article clearly shows how leucosceptoside A and leucosceptoside B are important compounds with regards to some aspects. In fact, they have been found in different species belonging to several families and, even though they do not possess chemophenetic significance on their own, they are also endowed with several interesting pharmacological activities including antioxidant, anti-inflammatory, enzyme inhibitory and neuroprotective. However, searching for these phytochemical components of plants is still quite limited due to the several factors. This review article also aimed to be a stimulus to amplify studies on both phytochemistry and pharmacology since there is still a lot to be discovered. 

## Figures and Tables

**Table 1:** Occurrence of leucosceptoside A in the plant kingdom.

Leucosceptoside A					
Family	Plant Species	Collection Area	Organs	Methodology of Isolation and Identification	Reference
Acanthaceae Juss.	*Acanthus ebracteatus* Vahl	Thailand	Aerial parts	SE, DP, CC, p-HPLC-UV, NMR	[5]
		Thailand (**obtained from a botanical garden**)	Leaves	SE, UHPLC-MS	[6]
	*Acanthus hirsutus* Boiss.	Turkey (**obtained from a botanical garden**)	Aerial parts	SE, PP, CC, UV, IR, NMR, MS	[7]
	*Acanthus montanus* (Nees) T. Anderson	Thailand (**obtained from a botanical garden**)	Aerial parts	SE, PP, CC, p-HPLC-RID, NMR	[8]
	*Blepharis edulis* (Forssk.) Pers.	Egypt	Aerial parts	SE, VLC, CC, TLC, HPLC-UV, NMR, MS	[9]
	*Pseuderanthemum carruthersii* (Seem.) Guillaumin	n.a.	n.a.	n.a.	[10]
Asteraceae Giseke	*Balbisia calycina* Hunz. and Ariza Esp.	Paraguay (**purchased from a company**)	Leaves	SE, DP, CC, TLC, HPLC-UV, NMR, MS	[11]
Bignoniaceae Juss.	*Fernandoa adenophylla* (Wall. ex G.Don) Steenis	Thailand (**obtained from a botanical garden**)	Leaves and branches	SE, DP, CC, TLC, p-HPLC-UV, NMR	[12]
	*Incarvillea compacta* Maxim.	China	Roots	SE, PP, CC, rp-CC, sp-LC-UV, NMR, MS	[13]
	*Incarvillea emodi* (Royle ex Lindl.) Chatterjee	Pakistan (**several populations**)	Whole plant	SE, CC, HPLC-DAD	[14]
	*Martinella obovata* (Kunth) Bureau and K.Schum.	Honduras	Leaves	SE, CC, TLC, HPLC-RID, NMR, MS	[15]
	*Oroxylum indicum* (L.) Kurz	Vietnam	Stem bark	USE, PP, CC, rp-MPLC, NMR	[16]
	*Santisukia kerrii* (Barnett and Sandwith) Brummitt	Thailand (**obtained from a botanical garden**)	Leaves and branches	SE, DP, CC, TLC, p-HPLC-UV, NMR	[17]
	*Tynanthus panurensis* (Bureau ex Baill.) Sandwith	Peru	Bark	SE, CC, HPLC-DAD, NMR, MS	[18]
Lamiaceae Martinov	*Betonica macrantha* C. Koch.	Turkey	Aerial parts	SE, DP, PP, CC, IR, IV, NMR	[19]
	*Callicarpa longissima* (Hemsl.) Merr.	China	Leaves and stems	SER, CC, TLC, p-HPLC-UV, NMR	[20]
	*Callicarpa nudiflora* Hook. and Arn.	n.a.	n.a.	n.a.	[21]
	*Caryopteris incana* (Thunb. ex Houtt.) Miq.	Japan	Aerial parts	SE, PP, CC, DCCC, NMR	[22]
		South Korea (**obtained from a botanical garden**)	Aerial parts	SE, PP, CC, TLC, NMR	[23]
		Japan (**cultivated**)	Whole plant	SE, PP, CC, p-HPLC-UV, TLC, NMR	[24]
		South Korea	Leaves	SER, PP, CC, TLC, UV, IR, HPLC-UV, NMR	[25]
	*Clerodendrum bungei* Steud.	n.a.	n.a.	n.a.	[26]
		China	Roots	SER, PP, CC, sp-HPLC-UV, NMR	[27]
	*Clerodendrum chinense* (Osbeck) Mabb.	n.a.	n.a.	n.a.	[28]
	*Clerodendrum infortunatum* L.	Bangladesh	Leaves	SE, PP, CC, TLC, sp-HPLC-UV, NMR	[29]
	*Clerodendrum phlomidis* L. f.	India (**obtained from a botanical garden**)	Roots	SPE, PP, CC, HPTLC, NMR, MS	[30]
		India (**obtained from a botanical garden**)	Roots	SPE, PP, TLC, CC, p-TLC, NMR	[31]
	*Clerodendrum trichotomum* Thunb.	South Korea	Stems	SE, PP, CC, [α]_D_, UV, NMR, MS	[32]
		South Korea	Stems	SE, PP, CC, NMR	[33]
		n.a.	n.a.	n.a.	[34]
	*Comanthosphace japonica* (Miq.) S.Moore	Japan	Roots	HSE, PP, CC, [α]_D_, IR, UV, NMR	[35]
		Japan	Whole plant	HSE, PP, CC, rp-CC, p-LPLC-UV, p-HPLC-UV, NMR	[36]
	*Galeopsis bifida* Boenn.	Siberia (**several populations**)	Leaves	USE, SPE, HPLC-DAD-MS	[37]
		Siberia (**several populations**)	Flowers	USE, SPE, HPLC-DAD-MS	[37]
		Siberia (**several populations**)	Stems	USE, SPE, HPLC-DAD-MS	[37]
		Siberia (**several populations**)	Roots	USE, SPE, HPLC-DAD-MS	[37]
	*Lagopsis supina* (Steph. ex Willd.) Ikonn.-Gal.	China	Whole plant	SE, PP, CC, LC-UV, rp-HPLC-UV, NMR	[38]
		Mongolia (**obtained from a botanical garden**)	Whole plant	SE, HPLC-DAD, UHPLC-MS^n^	[39]
		Mongolia	Whole plant	SE, PP, FP, TLC, UHPLC-MS	[40]
	*Leonurus cardiaca* L.	Siberia	Aerial parts	HSE, PP, HPLC-UV	[41]
	*Leonurus deminutus* V.I.Krecz.	Siberia	Aerial parts	HSE, PP, CC, HPLC-UV, NMR, MS	[41]
	*Leonurus glaucescens* Bunge	Siberia	Aerial parts	HSE, PP, HPLC-UV	[41]
	*Leonurus mongolicus* V.I.Krecz. and Kuprian.	Siberia	Aerial parts	HSE, PP, HPLC-UV	[41]
	*Leonurus persicus* Boiss.	Turkey	Aerial parts	SE, PP, VLC, TLC, NMR, MS	[42]
	*Leonurus quinquelobatus* Gilib.	Siberia	Aerial parts	HSE, PP, HPLC-UV	[41]
	*Leonurus sibiricus* L.	Siberia	Aerial parts	HSE, PP, HPLC-UV	[41]
		Mongolia	Aerial parts	ASE, DP, PP, HPLC-MS	[43]
		Mongolia (**several populations**)	Aerial parts	ASE, PP, CC, sp-HPLC-UV, HPLC-DAD-MS	[44]
	*Leonurus tataricus* L.	Siberia	Aerial parts	HSE, PP, HPLC-UV	[41]
	*Marrubium alysson* L.	Egypt	Aerial parts	HSE, DP, CC, MPLC, NMR	[45]
	*Marrubium thessalum* Boiss. and Heldr.	Greece	Aerial parts	SE, VLC, CC, TLC, NMR	[46]
	*Marrubium velutinum* Sm	Greece	Aerial parts	SE, VLC, CC, rp-HPLC-UV	[47]
	*Marrubium vulgare* L.	India	Whole plant	SE, PP, CC, TLC, p-TLC, NMR	[48]
	*Phlomis armeniaca* Willd.	Turkey	Aerial parts	SE, PP, CC, TLC, NMR, MS	[49]
	*Phlomis bruguieri* Desf.	Turkey	Aerial parts	HSE, PP, CC, HPLC-PAD, HPLC-PAD-MS	[50]
	*Phlomis chimerae* Boissieu	n.a.	n.a.	n.a.	[51]
	*Phlomis fruticosa* L.	Montenegro	Aerial parts	SE, UHPLC-MS	[52]
	*Phlomis integrifolia* Hub.-Mor.	Turkey	Aerial parts	HSE, PP, CC, MPLC, TLC, IR, UV, NMR	[53]
		n.a.	n.a.	n.a.	[54]
	*Phlomis kurdica* Rech.f.	Turkey	Aerial parts	HSE, PP, CC, HPLC-PAD, HPLC-PAD-MS	[50]
	*Phlomis leucophracta* P.H.Davis and Hub.-Mor.	Turkey	Aerial parts	HSE, PP, CC, HPLC-PAD, HPLC-PAD-MS	[50]
	*Phlomis longifolia* Boiss. and Blanche	Turkey	Aerial parts	SE, PP, VLC, MPLC, CC, TLC, rp-MPLC, NMR	[55]
	*Phlomis nissolii* L.	Turkey	Aerial parts	SE, PP, CC, MPLC, HPLC, TLC, NMR, MS	[56]
		Turkey	Aerial parts	HSE, PP, CC, HPLC-PAD, HPLC-PAD-MS	[50]
	*Phlomis oppositiflora* Boiss. and Hausskn.	Turkey	Whole plant	SE, PP, CC, MPLC, TLC, NMR	[57]
	*Phlomis physocalyx* Hub.-Mor.	Turkey	Aerial parts	SE, CC, LPLC-UV, NMR	[58]
	*Phlomis russeliana* (Sims) Lag. ex Benth.	Turkey	Aerial parts	HSE, PP, CC, HPLC-PAD, HPLC-PAD-MS	[50]
	*Phlomis sieheana* Rech. F.	Turkey	Aerial parts	HSE, PP, CC, MPLC, TLC, NMR	[59]
	*Phlomis syriaca* Boiss.	Turkey	Aerial parts	HSE, PP, CC, MPLC, TLC, IR, UV, NMR, MS	[54]
	*Phlomis viscosa* Poir.	Turkey	Whole plant	SE, PP, CC, TLC, NMR, MS	[60]
	*Phlomoides glabra* (Boiss. ex Benth.) Kamelin and Makhm.	Iran	Rhizomes	SXE, VLC, p-TLC, p-rp-HPLC-UV, NMR, UV, MS	[61]
	*Phlomoides laciniata* (L.) Kamelin and Makhm.	Turkey	Aerial parts	HSE, DP, PP, rp-VLC, TLC, NMR	[62]
	*Phlomoides rotata* (Benth. ex Hook.f.) Mathiesen	China (**several populations**)	Stems	USE, UPLC-MS	[63]
		Tibet (**several collections**)	Aerial parts	USE, UPLC-MS	[64]
		Tibet (**several collections**)	Roots	USE, UPLC-MS	[64]
	*Phlomoides tuberosa* (L.) Moench	Turkey	Aerial parts	HSE, PP, CC, TLC, NMR, MS	[65]
	*Salvia digitaloides* Diels	China	Roots	SE, PP, CC, TLC, NMR	[66]
	*Salvia viridis* L.	England (**cultivated**)	Aerial parts	SE, PP, CC, TLC, HPLC-UV	[67]
		Poland (**obtained from a botanical garden**)	Aerial parts	HSE, UPLC-DAD, UPLC-DAD-MS	[68]
		Turkey	Roots	SXE, UHPLC-MS^n^	[69]
		Turkey	Roots	USE, UHPLC-MS^n^	[69]
		Turkey	Roots	MAE, UHPLC-MS^n^	[69]
		Turkey	Roots	SE, UHPLC-MS^n^	[69]
	*Schnabelia nepetifolia* (Benth.) P.D.Cantino	China	Whole plant	SE, PP, CC, MPLC, p-HPLC-UV, NMR	[70]
	*Scutellaria albida* subsp. *velenovskyi* (Rech.f.) Greuter and Burdet	Turkey	Whole plant	SE, MPLC, rp-HPLC-UV, NMR	[71]
	*Scutellaria baicalensis* Georgi	n.r.	n.r.	n.r.	[72]
		China (**purchased from a company**)	Roots	USE, UHPLC-UV-MS	[73]
		China (**several commercial samples**)	Roots	USE, UPLC-MS	[74]
		China (**purchased from a company**)	Roots	SER, UPLC-MS	[75]
	*Scutellaria edelbergii* Rech.f.	Pakistan	Whole plant	SE, PP, LC-MS	[76]
	*Scutellaria lateriflora* L.	Japan (**purchased from a company**)	Aerial parts	SE, DP, PP, CC, TLC, NMR	[77]
	*Scutellaria pinnatifida* A.Ham.	Turkey	Aerial parts	SE, PP, CC, TLC, IR, UV, NMR	[78]
	*Scutellaria prostrata* Jacquem. ex Benth.	Nepal	Roots	SE, TLC, GLC, NMR	[79]
	*Scutellaria salviifolia* Benth.	Turkey	Aerial parts	SE, PP, CC, TLC, NMR, MS	[49]
	*Sideritis cypria* Post	Cyprus (**cultivated**)	Flowers	SE, CC, TLC, p-TLC, NMR	[80]
		Cyprus (**cultivated**)	Leaves	SE, CC, TLC, p-TLC, NMR	[80]
		Cyprus (**cultivated**)	Aerial parts	SE, CC, TLC, p-TLC, NMR	[81]
	*Sideritis euboea* Heldr.	Greece (**cultivated**)	Aerial parts	SE, PP, VLC, CC, TLC, NMR	[82]
		Greece (**cultivated**)	Aerial parts	SE, CC, p-TLC, NMR	[83]
	*Sideritis lycia* Boiss. and Heldr.	Turkey	Aerial parts	SE, PP, CC, MPLC, TLC, UV, IR, NMR	[84]
	*Sideritis ozturkii* Aytaç and Aksoy	Turkey	Aerial parts	SE, CC, VLC, MPLC, TLC, NMR	[85]
	*Sideritis perfoliata* L.	Greece	Aerial parts	SXE, PP, VLC, CC, TLC, UV, NMR	[86]
		Turkey	Aerial parts	HP, SE, HPLC-DAD-MS^n^	[87]
	*Sideritis raeseri* Boiss. and Heldr.	Albania (**several populations**)	Aerial parts	HP, SE, HPLC-DAD-MS^n^	[87]
		Macedonia (**several populations**)	Aerial parts	HP, SE, HPLC-DAD-MS^n^	[87]
		Serbia (**cultivated**)	Aerial parts	SXE, HPLC-DAD, HPLC-MS	[88]
		Serbia (**several populations**)	Aerial parts	SXE, DP, CC, HPLC-DAD-MS	[89]
	*Sideritis scardica* Griseb.	Macedonia (**several populations**)	Aerial parts	HP, SE, HPLC-DAD-MS^n^	[87]
		Bulgaria	Aerial parts	HP, SE, HPLC-DAD-MS^n^	[87]
		Serbia (**several populations**)	Aerial parts	SXE, DP, CC, HPLC-MS	[89]
		Greece (**purchased from a company**)	Aerial parts	USE, UPLC-MS, HPLC-DAD	[90]
	*Sideritis sipylea* Boiss.	Greece	Aerial parts	HSE, CC, p-TLC, NMR	[91]
	*Sideritis syriaca* L.	Bulgaria	Aerial parts	HP, SE, HPLC-DAD-MS^n^	[87]
		Greece	Aerial parts	HP, SE, HPLC-DAD-MS^n^	[87]
	*Stachys affinis* Bunge	n.a.	n.a.	n.a.	[92]
		Japan (**cultivated**)	Leaves	SE, CC, NMR	[93]
		Italy (**cultivated**)	Tubers	SE, CC, NMR, MS	[94]
	*Stachys iva* Griseb.	Greece (**cultivated**)	Aerial parts	HSE, CC, p-TLC, NMR	[95]
	*Stachys lavandulifolia* Vahl	Azerbaijan	Aerial parts	SXE, SPE, p-rp-HPLC-UV, NMR, MS	[96]
	*Stachys rupestris* Montbret and Aucher ex Benth.	Turkey	n.r.	SE, HPLC-MS	[97]
	*Stachys tetragona* Boiss. and Heldr.	Greece	Aerial parts	SE, VLC, CC, rp-HPLC, NMR	[98]
	*Volkameria inermis* L.	Thailand (**obtained from a botanical garden**)	Aerial parts	SE, DP, CC, TLC, p-HPLC-UV, NMR	[99]
Linderniaceae Borsch, Kai Müll. & Eb.Fisch.	*Craterostigma plantagineum* Hochst.	Rwanda (**cultivated**)	Leaves	USE, HPLC-DAD-MS	[100]
	*Lindernia brevidens* Skan	Kenya (**cultivated**)	Leaves	USE, HPLC-DAD-MS	[100]
	*Lindernia subracemosa* De Wild.	Rwanda (**cultivated**)	Leaves	USE, HPLC-DAD-MS	[100]
Malvaceae Juss.	*Firmiana simplex* (L.) W.Wight	China	Roots	SE, PP, CC, TLC, NMR	[101]
Oleaceae Hoffmanns. & Link	*Osmanthus fragrans* Lour.	China	Leaves	n.r.	[102]
Orobanchaceae Vent.	*Orobanche aegyptiaca* Pers.	n.r.	n.r.	n.r.	[103]
	*Orobanche arenaria* Borkh.	Poland	Whole plant	ASE, SPE, rp-HPLC-UV, UHPLC-PDA-MS, sp-HPLC-PDA	[104]
	*Orobanche artemisiae-campestris* subsp. *picridis* (F. Schulz) O. Bolòs, Vigo, Masalles and Ninot	Poland	Whole plant	ASE, SPE, rp-HPLC-UV, UHPLC-PDA-MS, sp-HPLC-PDA	[104]
	*Orobanche caryophyllacea* Sm.	Poland	Whole plant	ASE, SPE, rp-HPLC-UV, UHPLC-PDA-MS, sp-HPLC-PDA	[104]
	*Orobanche caerulescens* K.Koch	Poland	Whole plant	ASE, SPE, rp-HPLC-UV, UHPLC-PDA-MS, sp-HPLC-PDA	[104]
	*Orobanche cernua* Loefl.	China	Whole plant	SER, HPLC-MS, NMR	[105]
		China	Whole plant	SE, PP, CC, sp-HPLC-UV, NMR, MS	[106]
	*Orobanche pycnostachya* Hance	n.r.	n.r.	n.r.	[103]
		China	Whole plant	SE, PP, CC, TLC, UV, NMR, MS	[107]
	*Euphrasia pectinata* Ten.	Turkey	Aerial parts	HSE, PP, VLC, CC, TLC, MPLC, NMR	[108]
		Turkey	Aerial parts	HSE, VLC, MPLC, IR, UV, NMR	[109]
	*Pedicularis acmodonta* Boiss.	n.a.	n.a.	n.a.	[110]
	*Pedicularis alaschanica* Maxim.	n.a.	n.a.	n.a.	[111]
		n.a.	n.a.	n.a.	[112]
	*Pedicularis albiflora* Prain	China	Whole plant	SE, PP, CC, TLC, NMR, MS	[113]
	*Pedicularis dolichocymba* Hand.-Mazz.	n.a.	n.a.	n.a.	[114]
	*Pedicularis kansuensis* Maxim.	Tibet	Whole plant	SER, PP, CC, TLC, ESP, NMR, MS	[115]
	*Pedicularis kerneri* Dalla Torre	Italy	Aerial parts	SE, CC, NMR, MS	[116]
	*Pedicularis longiflora* var. *tubiformis* (Klotzsch) Tsoong	China	Whole plant	SE, PP, CC, HSCCC, rp-HPLC-UV, NMR	[117]
	*Pedicularis nordmanniana* Bunge	Turkey	Aerial parts	SE, DP, CC, NMR	[118]
	*Pedicularis verticillata* L.	China	Whole plant	SE, PP, CC, TLC, NMR	[119]
	*Phtheirospermum japonicum* (Thunb.) Kanitz	Japan	Aerial parts	SE, PP, CC, TLC, p-HPLC-UV, [α]_D_, NMR	[120]
Plantaginaceae Juss.	*Globularia alypum* L.	Croatia	Aerial parts	HP, SER, HPLC-PDA-MS	[121]
		Croatia	Aerial parts	USE, PP, HPLC-PDA-MS	[122]
		Croatia	Aerial parts	SXE, PP, HPLC-PDA-MS	[122]
	*Globularia cordifolia* L.	Turkey	Underground parts	HSE, PP, VLC, MPLC, CC, NMR, MS	[123]
		Croatia	Aerial parts	HP, SER, HPLC-PDA-MS	[121]
		Croatia	Aerial parts	USE, PP, HPLC-PDA-MS	[122]
		Croatia	Aerial parts	SXE, PP, HPLC-PDA-MS	[122]
	*Globularia davisiana* O.Schwarz	Turkey	Aerial parts	HSE, PP, VLC, CC, TLC, rp-MPLC, NMR	[124]
	*Globularia meridionalis* (Podp.) O.Schwarz	Croatia	Aerial parts	HP, SER, HPLC-PDA-MS	[121]
		Croatia	Aerial parts	USE, PP, HPLC-PDA-MS	[122]
		Croatia	Aerial parts	SXE, PP, HPLC-PDA-MS	[122]
	*Globularia orientalis* L.	Turkey	Aerial parts	HSE, PP, VLC, MPLC, NMR	[125]
	*Globularia punctata* Lapeyr.	Croatia	Aerial parts	HP, SER, HPLC-PDA-MS	[121]
		Croatia	Aerial parts	USE, PP, HPLC-PDA-MS	[122]
		Croatia	Aerial parts	SXE, PP, HPLC-PDA-MS	[122]
	*Globularia sintenisii* Hausskn. and Wettst.	Turkey	Underground parts	SE, CC, MPLC, TLC, NMR	[126]
	*Lagotis brevituba* Maxim.	China (**purchased from a company**)	Whole plant	SE, PP, HPLC-UV-MS	[127]
	*Lagotis ramalana* Batalin	n.a.	n.a.	n.a.	[128]
	*Penstemon centranthifolius* (Benth.) Benth.	California	Aerial parts	SE, FCC, CC, TLC, NMR, MS	[129]
	*Penstemon crandallii* A. Nelson	Colorado	Leaves	SE, PP, VLC, NMR	[130]
	*Penstemon linarioides* A. Gray	New Mexico	Whole plant	SE, PP, CC, TLC, [α]_D_, UV, NMR, MS	[131]
	*Plantago asiatica* L.	Japan	Whole plant	HSE, CC, NMR	[132]
		China (**several populations**)	Seeds	HSE, UPLC-MS^n^	[133]
		China	Seeds	USE, UHPLC-MS	[134]
	*Plantago depressa* Willd.	China (**several populations**)	Seeds	HSE, UPLC-MS^n^	[133]
		China	Seeds	USE, UHPLC-MS	[134]
	*Plantago lanceolata* L.	Brazil (**purchased from a company**)	Aerial parts	SWE, HPLC-DAD-MS	[135]
	*Plantago major* L.	China	Seeds	USE, UHPLC-MS	[134]
		Brazil (**purchased from a company**)	Aerial parts	SWE, HPLC-DAD-MS	[135]
	*Plantago squarrosa* Murray	Egypt	Whole plant	PE, PP, TLC, CC, UV, IR, NMR, MS	[136]
	*Plantago subulata* L.	n.r.	Aerial parts	HSE, PP, CC, MPLC, UV, IR, NMR, MS	[137]
		Turkey	Aerial parts	HSE, PP, CC, MPLC, TLC, NMR	[138]
		Turkey	Roots	HSE, PP, CC, MPLC, TLC, NMR	[138]
	*Rehmannia glutinosa* (Gaertn.) DC.	Japan (**purchased from a company**)	Roots	SE, CC, HPLC-UV, TLC, NMR	[139]
		n.a.	n.a.	n.a.	[140]
		China (**purchased from a local market**)	Rhizomes	SE, PP, CC, TLC, NMR	[141]
		n.a.	n.a.	n.a.	[142]
		China	Roots	SER, UHPLC-MS	[143]
		China	Tuber root	SE, HPLC-UV, UHPLC-MS	[144]
		China (**cultivated**)	Leaves	SE, UHPLC-MS	[145]
		China (**cultivated**)	Tubers	SE, UHPLC-MS	[145]
		Vietnam	Roots	HSE, PP, DP, CC, TLC, NMR	[146]
	*Russelia equisetiformis Schltdl. and Cham.*	Japan	Aerial parts	SE, PP, CC, TLC, DCCC, HPLC-UV, NMR	[147]
Scrophulariaceae Juss.	*Buddleja davidii* Franch.	China	Roots	SER, PP, CC, TLC, p-HPLC-UV, NMR	[148]
	*Buddleja lindleyana* Fortune	China	Powder	SE, PP, CC, rp-CC, NMR	[149]
	*Buddleja officinalis* Maxim.	China	Flower buds	SE, PP, CC, TLC, NMR	[150]
	*Scrophularia umbrosa* L.	China	Whole plant	SER, PP, CC, sp-rp-HPLC-UV, NMR	[151]
	*Verbascum thapsus* L.	Italy (**cultivated**)	Leaves	SE, HPLC-DAD-MS, NMR	[152]
Verbenaceae J.St.-Hil.	*Aloysia citriodora* Palau	Spain	Commercial extract	HPLC-UV, HPLC-MS	[153]
		Peru (**purchased from a company**)	Aerial parts	SER, PP, CC, HPLC-UV, NMR	[154]
		Spain (**commercial extract**)	Leaves	SE, sp-HPLC-UV, rp-HPLC-DAD- MS	[155]
		Spain (**commercial extract**)	Leaves	SE, HPLC-UV, HPLC-MS, sp-HPLC-UV	[156]
	*Citharexylum flexuosum* (Ruiz and Pav.) D.Don	Tunisia (**obtained from a botanical garden**)	Trunk bark	SE, CC, p-HPLC-UV, NMR	[157]
	*Lippia alba* (Mill.) N.E.Br. ex Britton and P.Wilson	Brazil (**different chemotypes**)	Leaves	HSE, HPLC-DAD-MS	[158]
	*Phyla canescens* (Kunth) Greene	Japan (**obtained from a botanical garden**)	Aerial parts	SE, PP, CC, HPLC-UV, NMR, MS	[159]
	*Stachytarpheta cayennensis* (Rich.) Vahl	Panama	Whole plant	SE, PP, CC, p-TLC, NMR, MS	[160]
	*Stachytarpheta schottiana* Schauer	Brazil (**obtained from a botanical garden**)	Aerial parts	SE, DP, LC-MS	[161]
	*Verbena brasiliensis* Vell.	Japan	Aerial parts	SE, PP, CC, HPLC, NMR	[162]
	*Verbena hastata L.*	Canada (**purchased from a company**)	Whole plant	SE, DP, CC, TLC, pHPLC, NMR	[163]

[α]_D_: optical rotation; ASE = accelerated solvent extraction; CC: column chromatography; DCCC: droplet counter current chromatograph; DP: defatting procedure; ESP: procedure to eliminate sugars; FCC: flash column chromatography; FP = fraction purification; GLC: gas liquid chromatography; HP = homogenization procedure; HPLC-DAD: high pressure liquid chromatography coupled to diode array detector; HPLC-DAD-MS: high pressure liquid chromatography coupled to diode array detector and mass spectrometry; HPLC-DAD-MS^n^: high pressure liquid chromatography coupled to tandem mass spectrometry; HPLC-MS: high pressure liquid chromatography coupled to mass spectrometry; HPLC-PAD: high pressure liquid chromatography coupled to pulsed amperometry detector; HPLC-PAD-MS: high pressure liquid chromatography coupled to pulsed amperometry detector and mass spectrometry; HPLC-UV: high pressure liquid chromatography coupled to ultraviolet detector; HPLC-UV-MS: high pressure liquid chromatography coupled to ultraviolet detector and mass spectrometry; HPTLC: high performance thin layer chromatography; HSE: hot solvent extraction; HSCCC = high-performance countercurrent chromatography; IR: infrared spectroscopy; LC-MS: liquid chromatography coupled to mass spectrometry; LC-UV: liquid chromatography coupled to ultraviolet detector; LPLC-UV: preparative low pressure liquid chromatography coupled to ultraviolet detector; MAE = microwave-assisted extraction; MPLC: medium pressure liquid chromatography; MS: mass spectrometry; NMR: nuclear magnetic resonance spectroscopy; n.a. = not accessible; n.r. = not reported; PE = percolation procedure; p-HPLC: preparative high pressure liquid chromatography; p-HPLC-RID: preparative high pressure liquid chromatography coupled to a refractive index detector; p-HPLC-UV: preparative high pressure liquid chromatography coupled to ultraviolet detector; p-LPLC-UV: preparative low pressure liquid chromatography coupled to ultraviolet detector; p-rp-HPLC-UV: preparative reversed-phase high pressure liquid chromatography coupled to ultraviolet detector; PP: partition procedure; p-TLC: preparative thin-layer chromatography; rp-CC: reversed-phase column chromatography; rp-HPLC-DAD-MS: reversed-phase high pressure liquid chromatography coupled to diode array detector and mass spectrometry; rp-HPLC-UV: reversed-phase high pressure liquid chromatography coupled to ultraviolet detector; rp-MPLC: reversed-phase medium pressure liquid chromatography; rp-VLC: reversed-phase vacuum liquid chromatography; SE: solvent extraction; SER: solvent extraction under reflux; SPE = solvent extraction via percolation; sp-HPLC-PDA: semipreparative high pressure liquid chromatography coupled to photodiode array detector; sp-HPLC-UV: semipreparative high pressure liquid chromatography coupled to ultraviolet detector; sp-LC-UV: semipreparative liquid chromatography coupled to ultraviolet detector; sp-rp-HPLC-UV: semipreparative reversed-phase high pressure liquid chromatography coupled to ultraviolet detector; SWE: subcritical water extraction; SXE: Soxhlet extraction; TLC: thin-layer chromatography; UHPLC-PDA-MS: ultra-high pressure liquid chromatography coupled to photodiode array detector and mass spectrometry; UHPLC-UV-MS: ultra-high pressure liquid chromatography coupled to ultraviolet detector and mass spectrometry; UHPLC-MS: ultra-high pressure liquid chromatography coupled to mass spectrometry; UHPLC-MS^n^: ultra-high pressure liquid chromatography coupled to tandem mass spectrometry; UPLC-DAD: ultra-pressure liquid chromatography coupled to diode array detector; UPLC-DAD-MS: ultra-pressure liquid chromatography coupled to diode array detector and mass spectrometry; UPLC-MS: ultra-pressure liquid chromatography coupled to mass spectrometry; UPLC-MS^n^: ultra-pressure liquid chromatography coupled to tandem mass spectrometry; USE = solvent extraction under ultrasonic action; UV: ultraviolet spectroscopy; VLC: vacuum liquid chromatography.

**Table 2:** Occurrence of leucosceptoside B in the plant kingdom.

Leucosceptoside B					
Family	Plant Species	Collection Area	Organs	Methodology of Isolation and Identification	Reference
Bignoniaceae Juss.	*Amphilophium crucigerum* (L.) L.G.Lohmann	Panama	Stems	SE, PP, MPLC, LPLC-UV, NMR	[164]
Lamiaceae Martinov	*Callicarpa kwangtungensis* Chun	n.a.	n.a.	n.a.	[165]
	*Callicarpa macrophylla* Vahl	China	Whole plant	SE, PP, CC, sp-rp-HPLC-UV, NMR	[166]
	*Comanthosphace japonica* (Miq.) S.Moore	Japan	Roots	HSE, PP, CC, [α]_D_, IR, UV, NMR	[35]
	*Marrubium alysson* L.	Egypt	Aerial parts	HSE, DP, CC, MPLC, NMR, MS	[45]
	*Phlomis bovei* Noë	Algeria	Roots	VLC, CC, MPLC, rp-MPLC, NMR, MS	[167]
	*Phlomis herba-venti* subsp. *pungens* (Willd.) Maire ex DeFilipps	n.a.	n.a.	n.a.	[168]
		Turkey	Aerial parts	HSE, PP, CC, rp-MPLC, NMR	[169]
		Azerbaijan	Aerial parts	SE, PP, CC, UV, IR, NMR, MS	[170]
	*Phlomis kotschyana* Hub.-Mor.	Turkey	Aerial parts	HSE, PP, CC, rp-MPLC, UV, IR, NMR	[171]
	*Phlomis kurdica* Rech.f.	Jordan	Aerial parts	SE, CC, rp-HPLC-UV, NMR	[172]
		Turkey	Aerial parts	HSE, PP, CC, HPLC-PAD, HPLC-PAD-MS	[50]
	*Phlomis lycia* D.Don	Turkey	Aerial parts	HSE, PP, CC, MPLC, TLC, IR, UV, NMR, MS	[173]
	*Phlomis nissolii* L.	Turkey	Aerial parts	SE, PP, CC, MPLC, HPLC-UV, TLC, NMR, MS	[56]
		Turkey	Aerial parts	HSE, PP, CC, HPLC-PAD, HPLC-PAD-MS	[50]
	*Phlomis russeliana* (Sims) Lag. ex Benth.	Turkey	Aerial parts	HSE, PP, CC, HPLC-PAD, HPLC-PAD-MS	[50]
	*Phlomis viscosa* Poir.	Turkey	Whole plant	SE, PP, CC, MPLC, TLC, NMR, MS	[60]
	*Phlomoides rotata* (Benth. ex Hook.f.)	China	Whole plant	SER, PP, CC, HSCCC, HPLC-UV	[174]
	*Phlomoides umbrosa* (Turcz.) Kamelin and Makhm.	South Korea	Roots	SE, PP, CC, TLC, rp-HPLC-UV, sp-HPLC-UV, NMR	[175]
	*Schnabelia nepetifolia* (Benth.) P.D.Cantino	China	Whole plant	SE, PP, CC, MPLC, HPLC-UV, LC-UV, NMR	[70]
	*Schnabelia tetradonta* (Y.Z.Sun) C.Y.Wu and C.Chen	China	Roots	SE, PP, CC, NMR	[176]
		n.a.	n.a.	n.a.	[177]
	*Stachys officinalis* (L.) Trevis.	Japan (**cultivated**)	Aerial parts	SE, CC, p-HPLC-UV, NMR	[178]
Orobanchaceae Vent.	*Pedicularis longiflora* var. *tubiformis* (Klotzsch) Tsoong	China	Whole plant	SE, PP, CC, HSCCC, rp-HPLC-UV, NMR	[117]
Plantaginaceae Juss.	*Lagotis brevituba* Maxim.	China (**purchased from a company**)	Whole plant	SE, PP, HPLC-UV-MS	[127]
Scrophulariaceae Juss.	*Buddleja davidii* Franch.	China	Roots	SER, PP, CC, p-HPLC-UV,	[148]
	*Buddleja lindleyana* Fortune	China	Powder	SE, PP, CC, rp-CC, NMR	[149]
	*Verbascum densiflorum* Bertol.	Bulgaria (**cultivated**)	Leaves	SE, HPLC-DAD, NMR	[179]
	*Verbascum nigrum* L.	Bulgaria (**cultivated**)	Leaves	SE, HPLC-DAD, NMR	[179]
	*Verbascum phlomoides* L.	Bulgaria (**cultivated**)	Leaves	SE, HPLC-DAD, NMR	[179]
	*Verbascum phoeniceum* L.	Bulgaria (**cultivated**)	Leaves	SE, HPLC-DAD, NMR	[179]
	*Verbascum thapsus* L.	Japan (**obtained from a botanical garden**)	Whole plant	SE, CC, NMR	[180]
		Italy (**cultivated**)	Leaves	SE, HPLC-DAD-MS, NMR	[152]
	*Verbascum wiedemannianum* Fisch. and C.A.Mey.	Turkey	Roots	SER, VLC, MPLC, CC, TLC, [α]_D_, NMR, MS	[181]
	*Verbascum xanthophoeniceum* Griseb.	Bulgaria (**several populations**)	Aerial parts	SE, PP, CC, rp-HPLC-UV, NMR	[182]
		Bulgaria (**cultivated**)	Leaves	SE, HPLC-DAD, NMR	[179]
		Bulgaria (**several populations**)	Whole plant	SE, PP, CC, LC-MS, NMR	[183]
Verbenaceae J.St.-Hil.	*Lantana camara* L.	Egypt (**obtained from a botanical garden**)	Leaves	SE, DP, PP, HPLC-MS	[184]

[α]_D_: optical rotation; CC: column chromatography; DP: defatting procedure; HPLC-DAD: high pressure liquid chromatography coupled to diode array detector; HPLC-DAD-MS: high pressure liquid chromatography coupled to diode array detector and mass spectrometry; HPLC-MS: high pressure liquid chromatography coupled to mass spectrometry; HPLC-PAD: high pressure liquid chromatography coupled to pulsed amperometry detector; HPLC-PAD-MS: high pressure liquid chromatography coupled to pulsed amperometry detector and mass spectrometry; HPLC-UV: high pressure liquid chromatography coupled to ultraviolet detector; HPLC-UV-MS: high pressure liquid chromatography coupled to ultraviolet detector and mass spectrometry; HSE: hot solvent extraction; HSCCC = high-performance countercurrent chromatography; IR: infrared spectroscopy; LC-MS: liquid chromatography coupled to mass spectrometry; LC-UV: liquid chromatography coupled to ultraviolet detector; LPLC-UV: preparative low pressure liquid chromatography coupled to ultraviolet detector; MPLC: medium pressure liquid chromatography; NMR: nuclear magnetic resonance spectroscopy; MS: mass spectrometry; p-HPLC-UV: preparative high pressure liquid chromatography coupled to ultraviolet detector; PP: partition procedure; rp-CC: reversed-phase column chromatography; rp-HPLC-UV: reversed-phase high pressure liquid chromatography coupled to ultraviolet detector; rp-MPLC: reversed-phase medium pressure liquid chromatography; SE: solvent extraction; SER: solvent extraction under reflux; sp-HPLC-UV: semipreparative high pressure liquid chromatography coupled to ultraviolet detector; sp-rp-HPLC-UV: semipreparative reversed-phase high pressure liquid chromatography coupled to ultraviolet detector; TLC: thin-layer chromatography; UV: ultraviolet spectroscopy; VLC: vacuum liquid chromatography.

**Table 3:** Summary of the biological activities and efficacy values of leucosceptoside A.

Leucosceptoside A				
Biological Activity	Studied Element	Specific Methodology and/or Studied Cells	Efficacy Value	Reference
Antioxidant	DPPH.^+^		IC_50_ = 18.43 µg/mL	[7]
			IC_50_ = 53.32 µM	[13]
			IC_50_ = 76.0 µM	[58]
			IC_50_ = 125.4 µM	[60]
			IC_50_ = 72.14 µM	[150]
			EC_50_ = 11.26 µM	[117]
			EC_50_ = 25.7 µM	[154]
			RC_50_ = 0.0148 µg/mL	[61]
			Inhibition % = 41.8% (at the concentration of 200 µM)	[54]
			- Inhibition % = 88.3% (after 20 min at the concentration of 0.1 mM) - Inhibition % = 89.0% (after 60 min at the concentration of 0.1 mM)	[86]
Radical scavenging	Superoxide anion	Luminol-enhanced chemiluminescence assay in stimulated human polymorphonuclear neutrophils	SC_50_ = 0.294 mM	[112]
	Hydroxyl radical	Luminol-enhanced chemiluminescence assay in stimulated human polymorphonuclear neutrophils	Inhibition % = 27.8% (at the concentration of 0.55 mM)	[112]
	Iron reductor	Luminol-enhanced chemiluminescence assay in stimulated human polymorphonuclear neutrophils	Inhibition % = 17.86% (at the concentration of 1.57 mM)	[112]
	*N*-formyl-methionyl-leucyl-phenylalanine	Luminol-enhanced chemiluminescence assay in stimulated human polymorphonuclear neutrophils	IC_50_ = 0.18 µM	[185]
Anti-inflammatory	NO production	Griess assay in LPS-stimulated BV2 microglial cells	IC_50_ = 61.1 µM	[14]
		Cell culture supernatants of LPS-stimulated RAW264.7 macrophages	Inhibition % = 40.0% (at the concentration of 100 µM)	[147]
		Raw 264.7 cells	IC_50_ = 9.0 µM	[151]
Enzyme inhibitory	A-glucosidase		IC_50_ = 0.7 mM	[27]
			IC_50_ = 273.0 µM	[146]
	Acetylcholinesterase		IC_50_ = 423.7 µg/mL	[33]
			IC_50_ = 72.85 µM	[186]
		Scheffe’s test	IC_50_ = 3.86 mM	[187]
	Protein kinase C alpha		IC_50_ = 19.0 µM	[131]
	Tyrosinase		- Inhibition % = 39.5% (at the concentration of 100 µM)	[186]
			- Inhibition % = 21.65% (at the concentration of 0.051 mM)	[188]
	Hyaluronidase		No activity	[138]
Hepatoprotective	CCl_4_ intoxication	MTT assay and flow cytometry in HepG2 cells	- Cell number % > 80% - Cell viability % > 80% - Inhibition % of CCl_4_-induced lipid peroxidation = 177.73% - SOD decrease attenuation % = 76.58%	[13]
Neuroprotective	1-methyl-4-phenylpyridinium ion (MPP^+^)-induced cell death	MTT assay in mesencephalic neurons of rats	- Cell death reduction % = 7% (at the concentration of 4 µM) - Cell growth increase % = 3.7% (at the concentration of 16 µM)	[189]
Cytoprotective	*t*-BHP-induced toxicity	HepG2 cells	IC_50_ = 21.1 µM	[23]
Anticomplementary			CH_50_ = 0.23 mM	[119]
Anti-HIV			IC_50_ = 29.4 µM	[32]
Cytotoxic	A549		IC_50_ = 99.00 µM	[186]
	B16F10		GI_50_ = 28 µM	[159]
	dRLh-88		No activity	[49]
	Hela		IC_50_ = 200 µg/mL	[46]
			IC_50_ = 80.87 µM	[186]
			GI_50_ = 42 µM	[159]
			No activity	[49]
	HC7-116		IC_50_ = 182.33 µg/mL	[46]
	MCF7		IC_50_ = 189.08 µg/mL	[46]
	MK-1		GI_50_ = 33 µM	[159]
	Melanoma		No activity	[46]
	P-388-d1		No activity	[49]
	S-180		No activity	[49]
Inhibitory	ADP + NADPH-induced lipid peroxidation	Rat liver microsome	IC_50_ = 1.69 µM	[132]
	Protonation of thymine radical anion induced by pulse radiolysis		Reaction rate constant = 1.54 × 10^9^ dm^3^ mol^−1^ s^−1^	[190]
Antimicrobial	*Staphylococcus aureus* ATCC29213		MIC = 1000 µg/mL	[60]
	*Enterococcus faecalis* ATCC29212		MIC = 1000 µg/mL	[60]
	*Bacillus subtilis* NBRC3134		No activity	[24]
	*Escherichia coli* ATCC25922		No activity	[60]
	*Klebsiella pneumoniae* NBRC3512		No activity	[24]
	*Mycobacterium tuberculosis* H37Rv		No activity	[30]
	*Pseudomonas aeruginosa* ATCC27853		No activity	[60]
Antifungal	*Candida albicans* ATCC90028		No activity	[60]
	*Candida krusei* ATCC6258		No activity	[60]
	*Candida parapsilosis* ATCC22019		No activity	[60]

**Table 4:** Summary of the biological activities and efficacy values of leucosceptoside B.

Leucosceptoside B				
Biological Activity	Studied Element	Specific Methodology and/or Studied Cells	Efficacy Value	Reference
Antioxidant	DPPH.^+^		IC_50_ = 61.3 µM	[166]
			IC_50_ = 31.16 µM	[166]
			IC_50_ = 96 µM	[182]
			EC_50_ = 13.05 µM	[117]
			EC_50_ = 25.7 µM	[154]
Radical scavenging	FRAP		Absorbance value = 0.602 (at 700 nm)	[183]
	HORAC		3885.1 HORACFL/g	[182]
	ORAC		16264.7 ORACFL/g	[182]
	Superoxide anion		IC_50_ = about 45 µg/mL	[182]
	*N*-formyl-methionyl-leucyl-phenylalanine	Luminol-enhanced chemiluminescence assay in stimulated human polymorphonuclear neutrophils	IC_50_ = 0.17 µM	[185]
Anti-inflammatory	Cobra venom factor induced alternative pathway activation	Mouse sera	Inhibition % = 40%	[183]
	COX-2 production		Inhibition % = about 30%	[183]
	IL-10 production		Value not reported	[183]
	NO production		Value not reported	[183]
Enzyme inhibitory	Acetylcholinesterase		inhibition % = about 50% (at the concentration of 100 µg/mL)	[182]
			IC_50_ = 20.1 µg/mL	[191]
	Butyrylcholinesterase		Inhibition % = a little above 10% (at the concentration of 100 µg/mL)	[182]
			No activity	[191]
Neuroprotective	1-methyl-4-phenylpyridinium ion (MPP^+^)-induced cell death	MTT assay in mesencephalic neurons of rats	Optical density value = 1.06 (at the concentration of 40 µg/mL)	[149]
Inhibitory	Human lactate dehydrogenase		No activity	[172]
Antimicrobial	*Staphylococcus aureus* ATCC29213		MIC = 1000 µg/mL	[60]
	*Enterococcus faecalis* ATCC29212		MIC = 1000 µg/mL	[60]
	*Bacillus subtilis* NBRC3134		No activity	[24]
	*Escherichia coli* ATCC25922		No activity	[60]
	*Pseudomonas aeruginosa* ATCC27853		No activity	[60]
Antifungal	*Candida albicans* ATCC90028		No activity	[60]
	*Candida krusei* ATCC6258		No activity	[60]
	*Candida parapsilosis* ATCC22019		No activity	[60]

## Data Availability

Not applicable.
